# Chronic unpredictable stress induces depression-related behaviors by suppressing AgRP neuron activity

**DOI:** 10.1038/s41380-020-01004-x

**Published:** 2021-01-11

**Authors:** Xing Fang, Shujun Jiang, Jiangong Wang, Yu Bai, Chung Sub Kim, David Blake, Neal L. Weintraub, Yun Lei, Xin-Yun Lu

**Affiliations:** 1 Department of Neuroscience & Regenerative Medicine, Medical College of Georgia at Augusta University, Augusta, GA USA; 2 Department of Physiology, Binzhou Medical University, Shandong, China; 3 Department of Medicine, Vascular Biology Center, Medical College of Georgia at Augusta University, Augusta, GA USA

**Keywords:** Depression, Neuroscience

## Abstract

Previous studies have shown that AgRP neurons in the arcuate nucleus (ARC) respond to energy deficits and play a key role in the control of feeding behavior and metabolism. Here, we demonstrate that chronic unpredictable stress, an animal model of depression, decreases spontaneous firing rates, increases firing irregularity and alters the firing properties of AgRP neurons in both male and female mice. These changes are associated with enhanced inhibitory synaptic transmission and reduced intrinsic neuronal excitability. Chemogenetic inhibition of AgRP neurons increases susceptibility to subthreshold unpredictable stress. Conversely, chemogenetic activation of AgRP neurons completely reverses anhedonic and despair behaviors induced by chronic unpredictable stress. These results indicate that chronic stress induces maladaptive synaptic and intrinsic plasticity, leading to hypoactivity of AgRP neurons and subsequently causing behavioral changes. Our findings suggest that AgRP neurons in the ARC are a key component of neural circuitry involved in mediating depression-related behaviors and that increasing AgRP neuronal activity coule be a novel and effective treatment for depression.

## Introduction

Major depression is one of the most common mental disorders. The lifetime prevalence of depression is 21% among American adults [1], with women having about twice the risk of developing the condition as men. Only about one-third of patients with major depression can achieve full remission with available treatments. Major depression can cause a variety of symptoms, but anhedonia is one of the two core symptoms required for the diagnosis [2–4]. Individuals can experience one or multiple types of anhedonia, defined as loss of interest or pleasure [5, 6]. Anhedonia typically does not respond to first-line pharmacotherapies [5, 7, 8] and is usually the last symptom to resolve in depression [9]. It has also been considered as a robust predictor of suicidality [10, 11]. Despite extensive research, the mechanisms underlying the development of hedonic deficits and other key symptoms of depression remain poorly understood.

The “classic” emotion-related brain structures, e.g., the prefrontal cortex and hippocampus, have attracted major attention as neural substrates underlying depression and treatment responses [12–15]. Given the high proportion of refractory or treatment-resistant depression, there is an urgent need for a better understanding of the pathogenesis of depression and the development of new antidepressants with novel mechanisms of action. We and others have previously shown that neurons in the arcuate nucleus (ARC) of the hypothalamus are activated by emotional stimuli [16, 17]. These neurons are well known to be involved in feeding and food reward processing [18, 19]. Neurons expressing agouti-related protein (AgRP) are found exclusively in the ARC [20], and they are stimulated by hunger signals [21, 22] and inhibited by overfeeding and satiety signals [23–25]. Optogenetic and chemogenetic activation of AgRP neurons can evoke feeding in fed mice and promote motivationally, food-seeking behavior [26, 27]. Conversely, inhibition of these neurons suppresses feeding [27]. Paradoxically, ablation of AgRP neurons has been reported to enhance isolation stress-induced anorexia and palatability-driven feeding [28]. We have previously demonstrated that AgRP neurons can respond rapidly to acute emotional stress [16]. However, a recent study failed to show a change in AgRP neuron activity after repeated restraint stress [29]. These findings suggest that AgRP neurons are involved in the complex interaction of stress, food reward, and ingestive behavior. However, their functional roles in determining the susceptibility to stress and the pathogenesis of depression remain unknown.

The preclinical research on depression has relied on valid animal models. As chronic stress increases the risk of developing depression in humans [30], it is not surprising that the vast majority of animal models are based upon exposure to different types of stress [31–33]. Chronic unpredictable/mild/variable stress and chronic social defeat stress are the two most commonly used animal models of depression [31, 34–38]. While the chronic social defeat stress model also shows features of post-traumatic stress disorder [39], chronic unpredictable/mild/variable stress induces a myriad of behavioral, neurochemical, and neuroendocrine alterations that mirror changes mainly observed in human depression [38]. One major advantage of the chronic unpredictable/mild/variable stress model is that it allows us to compare the effects of stress between males and females. The induction of anhedonia has been the primary focus in this model, along with its important feature reproducing hypercortisolism, which is found in 40–60% of patients with major depression and has been the most consistent biological abnormality in this disorder [38, 40].

Therefore, a chronic unpredictable stress paradigm was used in the present study to investigate whether and how the activity of AgRP neurons is influenced by chronic stress exposure in the context of depression, and whether manipulating their activity affects stress susceptibility and the development of depression-related behaviors in both male and female mice. Unlike the chronic unpredictable/mild stress paradigms used in other studies [38, 41], we excluded food and water deprivation due to their direct effects on metabolism and AgRP neuron activity. Using this model, we examined the impact of chronic stress on firing rates and patterns of AgRP neurons and explored synaptic and intrinsic mechanisms underlying chronic stress-induced changes in AgRP neuron activity. Furthermore, we employed a chemogenetic approach to selectively inhibit or activate AgRP neurons and determined how altering AgRP neuron activity affects stress susceptibility and chronic stress-induced depression-related behaviors.

## Materials and methods

### Animals

Wild-type C57BL/6J, *AgRP-ires-Cre* mice (Stock no. 012899), and *Ai14* mice (Stock no. 007914) were purchased from Jackson Laboratory (Bar Harbor, ME, USA). *AgRP-ires-Cre* knock-in mice have IRES-Cre inserted downstream of the stop codon of the Agrp locus [42]. *Ai14* mice have a loxP-flanked STOP cassette preventing transcription of a CAG promoter-driven tdTomato protein in all cells. *Ai14* mice express robust tdTomato fluorescence following Cre-mediated recombination [43]. For electrophysiological studies, *AgRP-ires-Cre* male mice were crossed with *Ai14* tdTomato female mice to obtain *AgRP-ires-Cre, Ai14* mice with tdTomato fluorescence in Cre-expressing cells (*Agrp-ires-Cre;tdTomato*), which was used to identify AgRP neurons. Animals were housed in groups of 3–5 under a 12/12-h light/dark cycle (lights on at 0600 h) with ad libitum access to water and standard food pellets. Both male and female mice at an age between 7 and 11 weeks were used, and all animal procedures were approved by the Institutional Animal Care and use Committees of the University of Texas Health Science Center at San Antonio and Augusta University.

### Viral injection

Male and female *AgRP-ires-Cre* mice at 7 weeks of age were anesthetized with an intraperitoneal (i.p.) injection of a cocktail containing xylazine (10 mg/kg) and ketamine (100 mg/kg) diluted in saline and then mounted onto a stereotaxic frame, as described elsewhere [36, 44]. Cre-dependent AAV5-hSyn-DIO-hM3D(Gq)-mCherry (subsequently referred to as AAV-DIO-hM3Dq-mCherry; titer, 8.2 × 10^12^ viral genomes per ml), AAV5-hSyn-DIO-hM4D(Gi)-mCherry (referred to as AAV-DIO-hM4Di-mCherry; titer, 5.3 × 10^12^ viral genomes per ml), and AAV5-hSyn-DIO-mCherry (referred to as AAV-DIO-mCherry; titer, 1.8 × 10^12^ viral genomes per ml), a gift from Bryan Roth (Addgene viral prep # 44361-AAV5, # 50475-AAV5, and # 50459-AAV5, respectively) [27], were injected into the ARC (coordinates: AP −1.4 mm, ML ± 0.2 mm, DV −5.8 mm from bregma). A volume of 0.2 μL AAV vectors was delivered bilaterally into the ARC at a rate of 0.1 μL/min with a 33-gauge stainless steel injector connected to a UMP3 microsyringe pump (World Precision Instruments, Sarasota, FL). Additional 5 min were allowed for diffusion and prevention of backflow. Behavioral procedures were conducted 14 days after AAV injection. The injection sites were verified by examining mCherry fluorescence in each animal at the end of the experiments. The animals with mis-injections were excluded from statistical analysis.

### Whole-cell patch-clamp recordings

Electrophysiological recordings were performed as previously described [36, 45]. Control and chronically stressed mice (8–10 weeks old) were anesthetized with isoflurane, and brains were quickly removed and transferred to an ice-cold cutting solution (254 mM sucrose, 3 mM KCl, 2 mM MgCl_2_, 2 mM CaCl_2_, 1.25 mM NaH_2_PO_4_, 10 mM D-glucose, and 24 mM NaHCO_3_). A tissue block containing the hypothalamus was immediately dissected. Coronal brain slices (~300 μm) were prepared with a Leica VT1000S Vibratome (Leica Microsystems), equilibrated at 32 °C for 30 min and subsequently maintained at room temperature for another 30 min in an oxygenated (95% O_2_/5% CO_2_) artificial cerebrospinal fluid solution (aCSF; 124 mM NaCl, 2 mM KCl, 2 mM MgSO_4_, 2 mM CaCl_2_, 1.25 mM NaH_2_PO_4_, 26 mM NaHCO_3_, and 10 mM Glucose, with pH 7.3 and osmolarity 300 mOsm) prior to recordings. Slices were transferred to the recording chamber and superfused in carbogenated aCSF at a flow rate of 2 ml/min at room temperature.

Neurons were visualized with a fixed stage upright microscope (Examiner.A1, Zeiss, NY) using 5× and 40× water-immersion objectives with infrared differential interference contrast optics (IR-DIC) and fluorescence optics. AgRP neurons were identified by their anatomical location in the hypothalamus and by their fluorescence that was visualized with Calibri.2 illumination system (Zeiss) in combination with a filter. Patch electrodes (3–5 MΩ) were prepared with capillary glass (external diameter 1.5 mm and internal diameter 0.86 mm, Sutter Instrument, CA) using a Flaming/Brown micropipette puller (P-97, Sutter Instrument, CA) and filled with a potassium gluconate-based internal solution (120 mM potassium gluconate, 20 mM KCl, 2 mM MgCl_2_, 10 mM HEPES, 2 mM ATP, 0.25 mM GTP and 0.1 mM EGTA adjusted to 7.4 and osmolarity of 295 mOsm). All recordings were made using a MultiClamp 700B microelectrode amplifier (Molecular Devices, LLC., CA), and data were filtered at 2 kHz and digitized at 10 kHz by using Axon Digidata 1550 A (Axon Instruments), and analyzed on a PC computer with pCLAMP 10.7 programs (Molecular Devices, LLC., CA). Membrane potential and spontaneous action potential (AP) firing rates were measured by using whole-cell current-clamp recording in the absence and presence of fast synaptic blockers, using 100 μM picrotoxin, 10 μM 6-cyano-7-nitroquinoxaline-2,3-dione (CNQX), and 50 μM DL-2-amino-5-phosphonopentanoic acid (DL-AP5) to inhibit GABA_A_ and ionotropic glutamate receptors AMPAR and NMDAR to isolate spontaneous, intrinsic AP. The interspike intervals (ISIs) for each neuron were measured from 3-min recordings; coefficient of variations (the ratio of the standard deviation of ISI to the mean of ISI) was calculated. For AP properties, the threshold was defined as the voltage where the first derivative of the voltage (dV/dt) exceeded 20 mV/ms [46]. AP amplitude and afterhyperpolarization (AHP) amplitude were measured from the resting membrane potential. AP duration, rise time, decay time, and half-width were determined using Clampfit 10.7 software (Axon Instruments). AP waveform was signal-averaged over three to five firing cycles for each neuron [47].

Spontaneous excitatory postsynaptic currents (EPSCs) and spontaneous inhibitory postsynaptic currents (IPSCs) were recorded from identified AgRP neurons in the voltage-clamp mode with membrane potentials held at −60 mV. Specifically, spontaneous EPSCs were recorded in the presence of 100 μM picrotoxin to eliminate ionotropic GABAergic transmission. Recordings of spontaneous IPSCs were made in the presence of AMPA and NMDA receptor antagonists (10 µM CNQX and 50 µM DL-AP5) to block glutamatergic responses. Tetrodotoxin (1 μM) was added to block AP formation and its propagation for the recording of miniature EPSCs and IPSCs. To analyze synaptic events, a template search was performed offline by Clampfit. Inter-event intervals and peak amplitudes of synaptic events were measured. The average amplitude and frequency of synaptic events were calculated at the end of the analysis for each neuron.

### Behavioral procedures

Adult male and female mice (9–11 weeks) were used for behavioral tests. Behavioral procedures were performed in the late light cycle except for the sucrose preference test, which was carried out during the first 2 h of the dark cycle. For behavioral tests involving chemogenetic activation or inhibition, mice received an i.p. injection of 0.3 mg/kg clozapine N-oxide (CNO; Sigma-Aldrich, Saint Louis, MO, USA) 30 min before testing. Behaviors were scored by the experimenters who were blinded to the treatments.

#### Chronic unpredictable stress

Mice (7–9 weeks old) were subjected to a variety of stressors at different times of the day for 10 days. The stressors included 2-h restraint, 15-min tail pinch, 24-h constant light, 24-h wet bedding with 45° cage tilt, 10-min inescapable foot shocks, 30-min elevated platform, and social isolation. Stress exposure was conducted in a procedure room, and mice exposed to the chronic unpredictable stress procedures were singly housed. Control mice were group-housed and briefly handled daily in the housing room.

#### Sucrose preference test

Mice were habituated to drinking from two bottles for 1 week before testing. Mice were separated into individual cages 4 h before the dark cycle with free access to food and received an i.p. injection of 0.3 mg/kg CNO 30 min prior to the dark cycle. The sucrose preference test was conducted during the first 2 h of the dark cycle, and during the test mice had free access to food, plain water, and 1% sucrose solution. Water and sucrose intake was measured, and the preference for sucrose was calculated by dividing the weight of sucrose intake consumed by the total weight of fluid intake.

#### Female urine sniffing test

This is a non-operant test to assess sex-related reward-seeking behavior based upon the interest of male rodents in pheromonal odors from estrus female urine [48]. Male mice were subjected to the following test procedure: (1) 3-min exposure to the cotton tip dipped in water; (2) a 45-min interval; (3) 3-min exposure to the cotton tip dipped in fresh urine collected from female mice in the estrus phase. The duration of female urine sniffing time was scored.

#### Forced swim test

Mice were placed in a clear Plexiglas cylinder (25 cm high; 10 cm in diameter) filled with 24 °C water to a depth of 15 cm. A charge-coupled device (CCD) camera positioned directly above the cylinder was used to record the behavior of each mouse for 6 min. The duration of immobility in the last 4 min was measured. Immobility was defined as no movement of the limb or body except those caused by respiration [35, 49, 50].

#### Locomotor activity

Mice were placed in SuperFlex Fusion open field cages (40 × 40 × 30 cm^3^, Omnitech Electronics Inc., OH) and allowed to freely explore for 30 min under illuminated conditions. The movements of mice were monitored by infrared photosensors equipped on the cage, and the total distance traveled was analyzed using the Fusion software (Omnitech Electronics Inc., OH).

### Immunohistochemistry

Immunohistochemistry was performed as described previously [51–53]. For c-Fos detection, mice injected with AAV-DIO-hM3Dq-mCherry or AAV-DIO-mCherry received one injection of CNO (0.3 mg/kg, i.p.), and 2 h later mice were transcardially perfused under anesthesia through the ascending aorta using 0.1 M phosphate-buffered saline (PBS) followed by 4% paraformaldehyde (PFA) in PBS. The brain was removed and post-fixed overnight in 4% PFA and then transferred to 30% sucrose in PBS. For detection of AgRP-positive neurons following multiple injections with CNO, mice received stereotaxic injection with AAV-DIO-hM3Dq-mCherry on one side of the ARC and the other side received the sham injection. Three weeks later, mice were injected with CNO (0.3 mg/kg per day, i.p.) for six consecutive days. Brain tissue was fixed as described above. Coronal brain tissues were cut (40-μm-thick) with a cryostat and stored in cryoprotectant (30% sucrose, 30% ethylene glycol, 1% polyvinyl pyrrolidone, 0.05 M sodium phosphate buffer) until processing for immunohistochemistry. Free-floating sections were first treated with 1% hydrogen peroxide in PBS to quench the endogenous peroxidase. The tissue was then incubated in a blocking buffer (3% goat serum, 1% bovine serum albumin, 0.3% triton-X 100 in PBS) for 1 h at room temperature followed by incubation with anti-c-Fos antibody (1:250, #sc-166940 X, Santa Cruz Biotechnology, TX) or anti-AgRP antibody (1:200, #H-003-57, Phoenix Pharmaceuticals, Inc., CA) in blocking solution for 48 h at 4 °C. After rinsing in PBS buffer, the sections were incubated with donkey anti-mouse secondary antibody (1:750, #A-21202, ThermoFisher Scientific, MA) or donkey anti-rabbit secondary antibody (1:750, #A-21206, ThermoFisher Scientific, MA) conjugated to Alexa Fluor 488 at room temperature for 4 h. NIKON A1R MP + multiphoton/confocal microscope was used to capture pictures.

### Statistical analysis

All results are presented as mean ± s.e.m. (standard error of the mean). Statistical analyses were performed with GraphPad Prism 8.0 (GraphPad Software, Inc., CA). Shapiro–Wilk test and *F* test were used to test the normality and equal variance assumptions, respectively. For normally distributed data, two-tailed *t*-tests were used to assess differences between two experimental groups with equal variances. For a two-sample comparison of means with unequal variances, two-tailed *t*-tests with the Welch’s correction were used. One-way analyses of variance (ANOVAs) followed by Bonferroni post hoc tests were used for the analysis of three or more groups. For non-normally distributed data, Mann–Whitney *U* tests were performed to compare two groups. For analysis of three or more groups with non-normal distribution, the Kruskal–Wallis test followed by Dunn’s multiple comparisons test was used. For the results of the female urine sniffing test, two-way ANOVAs followed by Bonferroni post hoc tests were applied for statistical analysis. Cumulative probabilities of amplitude and interevent interval distributions of postsynaptic currents were analyzed with the two-tailed Kolmogorov–Smirnov test. *P* < 0.05 was considered statistically significant.

## Results

### Chronic unpredictable stress induces anhedonia and despair behavior in both male and female mice

**Fig. 1:**
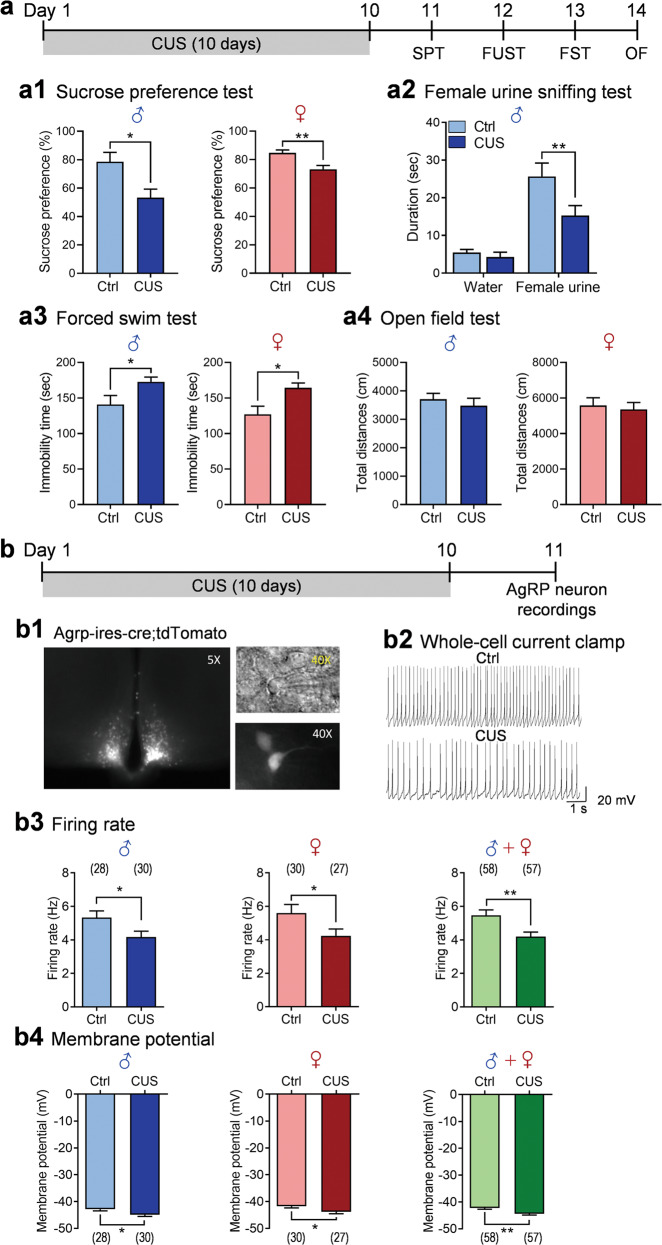

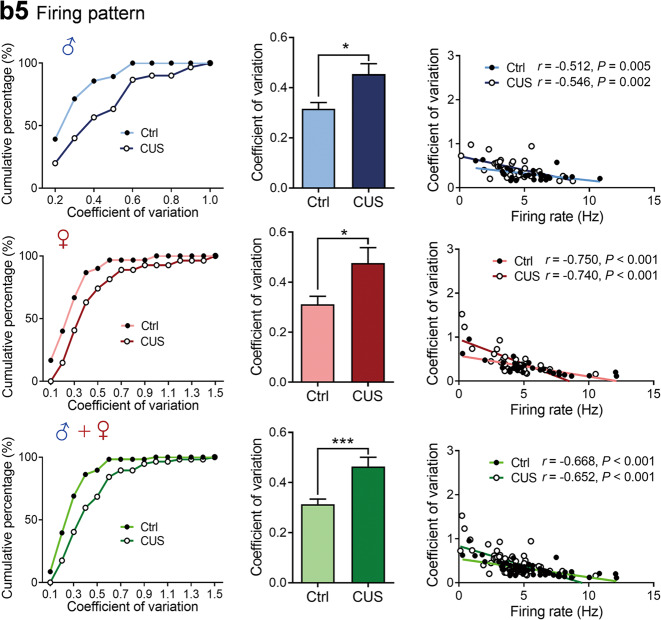
Chronic unpredictable stress induces depression-related behaviors and alters spontaneous firing rates and firing patterns of AgRP neurons. **a** Timeline of the chronic unpredictable stress (CUS) procedure and behavioral tests. SPT, sucrose preference test; FUST, female urine sniffing test; FST, forced swim test; OF, open field test. **a1** Sucrose preference test. **a2** Female urine sniffing test. **a3** Forced swim test. **a4** Open field test. Male mice: Ctrl, *n* = 9; CUS, *n* = 10; female mice: Ctrl, *n* = 10; CUS, *n* = 10. **b** Timeline of the CUS procedure and whole-cell current-clamp recordings of AgRP neurons. **b1** Left, representative fluorescent images of a coronal brain slice from an *Agrp-Cre;tdTomato* mouse showing fluorescent AgRP neurons in the arcuate nucleus (ARC); right, patch-clamp recording from a tdTomato-labeled AgRP neuron. **b2** Representative traces of spontaneous action potentials of AgRP neurons from control and CUS groups. **b3** Spontaneous firing rates. **b4** Membrane potential. **b5** Spontaneous firing patterns. Left, cumulative probability distributions of coefficients of variation; middle, average coefficients of variation; right, correlation analysis between spontaneous firing rates and coefficients of variation. Ctrl: *n* = 58 neurons from three male (28 neurons) and three female (30 neurons) mice. CUS: *n* = 57 neurons from three male (30 neurons) and three female (27 neurons) mice. *P < 0.05, **P < 0.01, ***P < 0.001 vs control group.

Our previous studies have shown that chronic unpredictable stress induces anhedonia and despair behavior in male mice [36]. To determine whether this stress paradigm can induce depression-related behaviors in both sexes, age-matched cohorts of male and female wild-type C57BL/6J mice were subjected to 10 days of unpredictable stress. To eliminate the effects of acute stress, behavioral testing began 24 h after the last stress exposure (Fig. 1a). The hedonic responses to sucrose reward were assessed using a two-bottle free-choice paradigm (1% sucrose vs water) during the first 2 h of the dark cycle with ad libitum access to food. We found that sucrose preference was reduced in both male and female mice after exposure to chronic unpredictable stress (Fig. 1a1; male: Mann Whitney test: *P* = 0.030; female: *t*_(18)_ = 3.365, *P* = 0.004), indicating hedonic deficits. To test whether this chronic unpredictable stress paradigm is able to induce anhedonic behavior irrelevant to food rewards, male mice were subjected to the female urine sniffing test, a test based upon the interest of male rodents in urine-born female pheromones [48]. Male mice exposed to chronic unpredictable stress spent less time sniffing female urine when compared with non-stressed controls, but no significant differences in time sniffing water were observed between the two groups (Fig. 1a2; stress: *F*_(1,34)_ = 6.191, *P* = 0.018; sniffing object: *F*_(1,84)_ = 45.15, *P* < 0.001; stress × sniffing object: *F*_(1,34)_ = 3.951, *P* = 0.055), suggesting a specific effect on sex-related reward-seeking behavior. Moreover, chronically stressed mice of both sexes exhibited increased immobility in the forced swim test without showing significant changes in locomotor activity measured in the open field (Fig. 1a3–a4; male: Mann Whitney test: *P* = 0.037; female: *t*_(18)_ = 2.811, *P* = 0.012; locomotor activity-male: *t*_(17)_ = 0.677, *P* = 0.508; locomotor activity-female: *t*_(18)_ = 0.374, *P* = 0.713).

### Chronic unpredictable stress suppresses the activity of AgRP neurons in both male and female mice

AgRP neurons are spontaneously active under control conditions [54]. To test whether chronic unpredictable stress modulates AgRP neuron firing, *Agrp*-*ires-Cre*;*tdTomato* reporter mice were used to perform whole-cell current-clamp recordings in tdTomato-labeled AgRP neurons in the ARC (Fig. 1b). Spontaneous firing activity of AgRP neurons was recorded 24 h following exposure to 10 days of unpredictable stress (Fig. 1b1, b2). We found that the firing rate was reduced (Fig. 1b3; male: *t*_(56)_ = 2.183, *P* = 0.033; female: *t*_(55)_ = 2.051, *P* = 0.045; total: *t*_(113)_ = 3.014, *P* = 0.003) and the membrane potential was hyperpolarized in both male and female mice after chronic unpredictable stress (Fig. 1b4; male: Mann Whitney test, *P* = 0.025; female: *t*_(55)_ = 2.212, *P* = 0.031; total: Mann Whitney test, *P* = 0.001). There were no sex differences in the firing rate or membrane potential under either control or chronic stress conditions (firing rate-Ctrl: *t*_(56)_ = 0.412, *P* = 0.682; firing rate-CUS: *t*_(55)_ = 0.135, *P* = 0.893; membrane potential-Ctrl: Mann Whitney test, *P* = 0.214; membrane potential-CUS: *t*_(55)_ = 1.184, *P* = 0.241).

Next, we examined the effect of chronic stress exposure on firing patterns of AgRP neurons, measured as the coefficient of variation of ISIs. A right shift of the cumulative frequency distribution of ISIs of different lengths (cumulative probability; male: Kolmogorov–Smirnov test, *P* = 0.052; female: Kolmogorov–Smirnov test, *P* = 0.128; total: Kolmogorov–Smirnov test, *P* = 0.0099) and an increase in firing irregularity (male: Mann Whitney test, *P* = 0.022; female: Mann Whitney test, *P* = 0.018; total: Mann Whitney test, *P* < 0.001) were observed in both male and female mice following exposure to chronic unpredictable stress (Fig. 1b5). Further analysis revealed that firing rates were negatively correlated with coefficients of variation of ISIs under control and chronic stress conditions (male-Ctrl: *r* = −0.512, *P* = 0.005; male-CUS: *r* = −0.546, *P* = 0.002; female-Ctrl: *r* = −0.750, *P* < 0.001; female-CUS: *r* = −0.740, *P* < 0.001; total-Ctrl: *r* = −0.668, *P* < 0.001; total-CUS: *r* = −0.652, *P* < 0.001); and mice exposed to chronic stress showed steeper slopes than control mice (Fig. 1b5). These results indicate that AgRP neurons from chronically stressed mice fire more slowly and irregularly than those from control mice.

### Chronic unpredictable stress increases inhibitory synaptic transmission in AgRP neurons

**Fig. 2:**
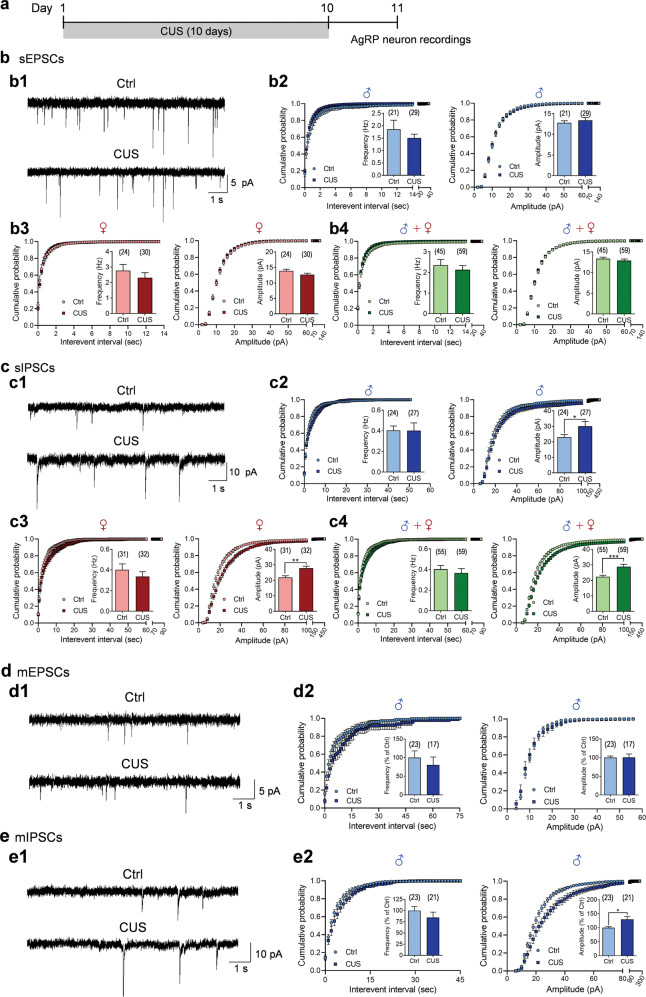
Effects of chronic unpredictable stress on synaptic plasticity of AgRP neurons. **a** Timeline of the chronic unpredictable stress (CUS) procedure and whole-cell voltage-clamp recordings of AgRP neurons. **b** Spontaneous excitatory postsynaptic currents (sEPSCs). **b1** Representative traces depicting sEPSCs recorded in AgRP neurons from control and CUS mice. **b2** Cumulative probability plots for the interevent interval (left) and amplitude (right) of sEPSCs recorded in AgRP neurons from male mice. The insets show the average frequency and amplitude of sEPSCs. **b3** Cumulative probability plots for the interevent interval (left) and amplitude (right) of sEPSCs recorded in AgRP neurons from female mice. The insets show the average frequency and amplitude of sEPSCs. **b4** Cumulative probability plots for the interevent interval (left) and amplitude (right) of sEPSCs recorded in AgRP neurons from mice of both sexes combined. The inserts show the average frequency and amplitude of sEPSCs. Control (Ctrl): *n* = 45 neurons from three male (21 neurons) and three female (24 neurons) mice. CUS: *n* = 59 neurons from three male (29 neurons) and three female (30 neurons) mice. **c** Spontaneous inhibitory postsynaptic currents (sIPSCs). **c1** Representative traces depicting sIPSCs recorded in AgRP neurons from control and CUS mice. **c2** Cumulative probability plots for the interevent interval (left) and amplitude (right) of sIPSCs recorded in AgRP neurons from male mice. The insets show the average frequency and amplitude of sIPSCs. **c3** Cumulative probability plots for the interevent interval (left) and amplitude (right) of sIPSCs recorded in AgRP neurons from female mice. The insets show the average frequency and amplitude of sIPSCs. **c4** Cumulative probability plots for the interevent interval (left) and amplitude (right) of sIPSCs recorded in AgRP neurons from mice of both sexes combined. The insets show the average frequency and amplitude of sIPSCs. Ctrl: *n* = 55 neurons from three male (24 neurons) and three female (31 neurons) mice. CUS: *n* = 59 neurons from three male (27 neurons) and three female (32 neurons) mice. **d** Miniature excitatory postsynaptic currents (mEPSCs). **d1** Representative traces depicting the mEPSCs recorded in AgRP neurons from control and CUS male mice. **d2** Cumulative probability plots for the interevent interval (left) and amplitude (right) of mEPSCs recorded in AgRP neurons from male mice. The insets show the average frequency and amplitude of mEPSCs. Ctrl: *n* = 23 neurons from three male mice. CUS: *n* = 17 neurons from three male mice. **e** Miniature inhibitory postsynaptic currents (mIPSCs). **e1** Representative traces depicting the mIPSCs recorded in AgRP neurons from control and CUS male mice. **e2** Cumulative probability plots for the interevent interval (left) and amplitude (right) of mIPSCs recorded in AgRP neurons from male mice. The insets show the average frequency and amplitude of mIPSCs. Ctrl: *n* = 23 neurons from three male mice. CUS: *n* = 21 neurons from three male mice. *P < 0.05, **P < 0.01, ***P < 0.001 vs control group.

The effects of chronic unpredictable stress on the spontaneous firing of AgRP neurons could be mediated via changes in synaptic inputs. A decrease in excitatory synaptic transmission or an increase in inhibitory synaptic transmission would decrease AP firing. Therefore, we examined synaptic transmission at excitatory and inhibitory synapses of AgRP neurons 24 h after 10 days of unpredictable stress (Fig. 2a). Whole-cell voltage-clamp recordings in the presence of 100 μM picrotoxin, a GABA_A_ receptor antagonist used to block GABAergic synaptic transmission, revealed that neither the frequency (male: Mann Whitney test, *P* = 0.895; female: Mann Whitney test, *P* = 0.385; total: Mann Whitney test, *P* = 0.790) nor the amplitude (male: Mann Whitney test, *P* = 0.938; female: *t*_(52)_ = 1.740, *P* = 0.088; total: Mann Whitney test, *P* = 0.173) of spontaneous EPSCs of AgRP neurons was altered by chronic unpredictable stress (Fig. 2b1–b4). Recordings of spontaneous IPSCs in AgRP neurons were made in the presence of AMPA and NMDA receptor antagonists to block glutamatergic synaptic transmission. While the frequency of spontaneous IPSCs remained unaltered (male: Mann Whitney test, *P* = 0.313; female: Mann Whitney test, *P* = 0.337; total: Mann Whitney test, *P* = 0.209), the amplitude was increased (Fig. 2c1–c4; male: Mann Whitney test, *P* = 0.038; female: *t*_(61)_ = 3.347, *P* = 0.001; total: Mann Whitney test, *P* < 0.001) and the cumulative distribution of the amplitudes was right-shifted (male: Kolmogorov–Smirnov test, *D* = 0.2446, *P* < 0.001; female: Kolmogorov–Smirnov test, *D* = 0.4246, *P* < 0.001; total: Kolmogorov–Smirnov test, *D* = 0.2736, *P* < 0.001) in both sexes after chronic unpredictable stress (Fig. 2c2–c4). These results demonstrate that chronic unpredictable stress exerts differential effects on excitatory and inhibitory inputs onto AgRP neurons.

Spontaneous EPSCs and IPSCs consist of AP-dependent and AP-independent events. To determine whether the effects of chronic unpredictable stress on spontaneous postsynaptic currents in AgRP neurons are AP-dependent, mice were subjected to 10 days of unpredictable stress, and 24 h after the last stress exposure, spontaneous AP-independent EPSCs, and IPSCs (miniature EPSCs and IPSCs) were recorded in the presence of 1 µM tetrodotoxin in order to eliminate AP-dependent events. Chronic unpredictable stress had no effect either on the frequency or amplitude of miniature EPSCs (Fig. 2d1–d2; frequency, Mann Whitney test, *P* = 0.397; amplitude, unpaired *t*-test with the Welch’s correction, *P* = 0.945). However, the amplitude of miniature IPSCs was significantly increased in AgRP neurons with a right-shifted cumulative distribution (Fig. 2e1-e2; the cumulative probability of amplitude, Kolmogorov–Smirnov test, *D* = 0.4542, *P* < 0.001; amplitude, Mann Whitney test, *P* = 0.012), while the frequency remained unaltered (Mann Whitney test, *P* = 0.083). These results demonstrated that chronic unpredictable stress increased inhibitory synaptic transmission onto AgRP neurons.

### Chronic unpredictable stress alters the intrinsic firing rate, patterns, and shape of AgRP neurons

**Fig. 3:**
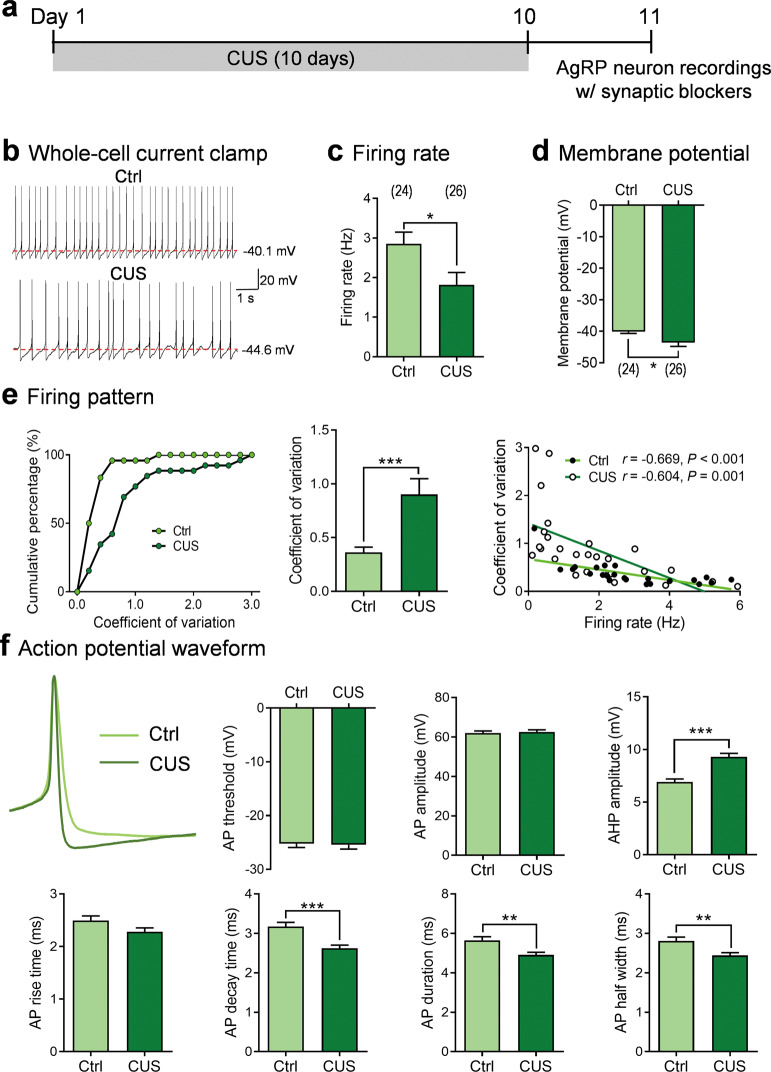
Chronic unpredictable stress alters intrinsic properties of AgRP neurons. **a** Experimental timeline. Whole-cell current-clamp recordings of AgRP neurons were performed in the presence of synaptic blockers. **b** Representative traces of intrinsic action potentials of AgRP neurons. **c** Firing rate. **d** Membrane potential. **e** Intrinsic firing patterns. Left, cumulative probability distributions of coefficients of variation; middle, average coefficients of variation; right, correlation between spontaneous firing rates and coefficients of variation. **f** Action potential (AP) properties. Upper panel: left, representative AP waveforms recorded in AgRP neurons from control and CUS mice; middle-left, AP threshold; middle-right, AP amplitude; right, afterhyperpolarization (AHP). Lower panel: left, AP rise time; middle-left, AP decay time; middle-right, AP duration; right, AP half-width. For each neuron, APs were signal-averaged from 3 to 5 firing cycles. Ctrl: *n* = 24 neurons from three mice (11 neurons from 2 male mice and 13 neurons from 1 female mouse); CUS: *n* = 26 neurons from three mice (17 neurons from 2 male mice and 9 neurons from 1 female mouse). *P < 0.05, ***P < 0.001.

Spontaneous firing patterns of individual neurons are regulated by both synaptic inputs and intrinsic excitability. It is possible that chronic unpredictable stress may modulate the intrinsic properties of AgRP neurons. Thus, we analyzed the effects of chronic unpredictable stress on intrinsic firing, e.g., the rate, pattern, and shape of AP, of AgRP neurons in the absence of excitatory and inhibitory synaptic inputs. Whole-cell current-clamp recordings were performed in AgRP neurons in the presence of fast synaptic blockers to inhibit GABA_A_ and ionotropic AMPA and NMDA glutamate receptors in order to isolate spontaneous, intrinsic APs. The intrinsic activity of AgRP neurons was clearly reduced in mice of both sexes after exposure to 10 days of unpredictable stress (Fig. 3a, b). The intrinsic firing frequency was decreased (Fig. 3b, c, Mann Whitney test, *P* = 0.011) and the membrane potential was hyperpolarized (Fig. 3d, unpaired *t*-test with the Welch’s correction, *P* = 0.014). Moreover, the percentage of AgRP neurons without spontaneous AP firing (silent neurons; firing rate <0.5 Hz) increased from 5% (1 of 24) in control mice to 23% (6 of 26) in chronically stressed mice. To examine the intrinsic firing pattern, we measured the coefficient of variation of ISIs. Chronic unpredictable stress caused a right shift of the cumulative frequency distribution of coefficient of variation of ISIs (Fig. 3e, Kolmogorov–Smirnov test, *P* < 0.001), and an increase in the irregularity of intrinsic firing of AgRP neurons (Fig. 3e, Mann Whitney test, *P* < 0.001). There was a negative correlation between intrinsic firing rates and coefficients of variation of ISIs under control and chronic stress conditions (Fig. 3e; Ctrl: *r* = −0.669, *P* < 0.001; CUS: *r* = −0.604, *P* = 0.001) with steeper slopes in chronically stressed mice than control mice (Fig. 3e). These results indicate that exposure to chronic unpredictable stress affects the frequency and patterns of intrinsic firing of AgRP neurons.

Furthermore, we analyzed whether chronic unpredictable stress affects the shape of APs. One component of the AP waveform, AHP, is important in the control of ISIs and firing frequency. Analysis of AP waveforms in AgRP neurons showed that chronic unpredictable stress had no significant effect on the threshold or amplitude of the AP (Fig. 3f; AP threshold: *t*_(48)_ = 0.206, *P* = 0.838; AP amplitude, *t*_(48)_ = 0.380, *P* = 0.706), but induced an increase in AHP (AHP amplitude, *t*_(48)_ = 5.482, *P* < 0.001). Moreover, AgRP neurons from chronically stressed mice exhibited unaltered rise time (Mann Whitney test, *P* = 0.073), but reduced decay time (*t*_(48)_ = 4.169, *P* = 0.0001), duration (Mann Whitney test, *P* = 0.002), and half-width (*t*_(48)_ = 3.148, *P* = 0.003) of APs (Fig. 3f). These data suggest that chronic unpredictable stress alters the kinetics of AP waveforms.

### Chemogenetic inhibition of AgRP neurons increases susceptibility to subthreshold unpredictable stress

**Fig. 4:**
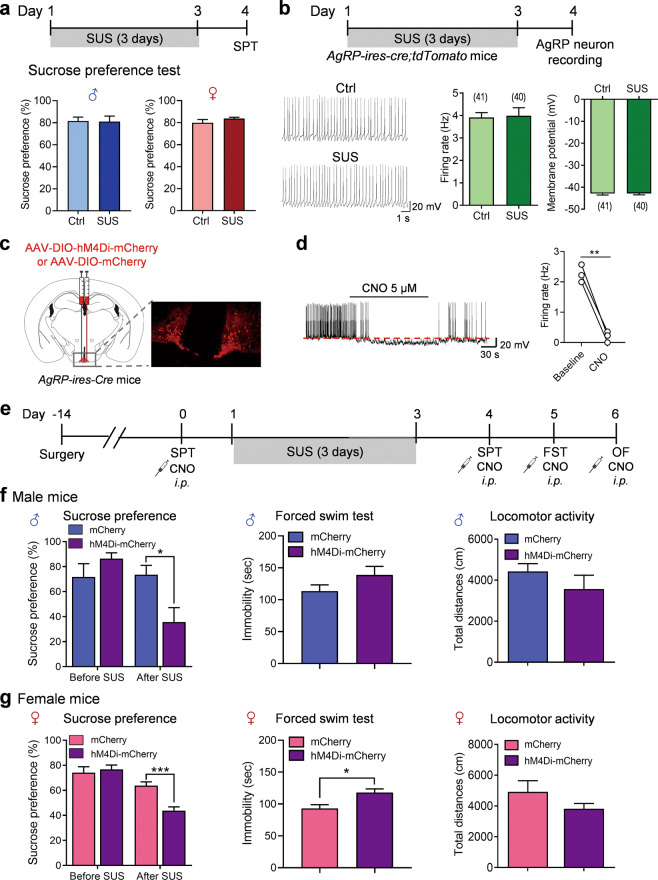
Chemogenetic inhibition of AgRP neurons increases susceptibility to subthreshold unpredictable stress. **a** Top, timeline of the subthreshold unpredictable stress (SUS) procedure and sucrose preference test (SPT). Bottom, sucrose preference test of male (left) and female (right) wild-type C57BL/6J mice. Male mice: Ctrl, *n* = 9; CUS, *n* = 9; female mice: Ctrl, *n* = 11; CUS, *n* = 10. **b** Top, a timeline of the SUS procedure and electrophysiological recordings in *Agrp-ires-Cre;tdTomato* mice; bottom-left, representative traces of spontaneous action potentials recorded in AgRP neurons from control and SUS groups; bottom-middle, firing rate; bottom-right, membrane potential. Ctrl: *n* = 41 neurons from three mice; SUS: *n* = 40 neurons from three mice. **c** Left, the schematic illustration showing stereotaxic injections of AAV-DIO-hM4Di-mCherry or AAV-DIO-mCherry in the arcuate nucleus (ARC) of *AgRP-ires-Cre* mice; right, a representative image of mCherry labeled AgRP neurons in the ARC. **d** Left, representative traces of action potentials recorded in AgRP neurons infected with AAV-DIO-hM4Di-mCherry in response to bath application of CNO (5 µM); right, firing rates before and after CNO application, *n* = 3 neurons. **e** Experimental timeline. **f** Left, sucrose preference test (SPT) of male mice in response to CNO injection (0.3 mg/kg, i.p.) before and after SUS; forced swim test (FST, middle) and locomotor activity in the open field (OF, right) of male mice in response to CNO injection after SUS. AAV-DIO-mCherry, *n* = 8; AAV-DIO-hM4Di-mCherry, *n* = 9. **g** Left, sucrose preference test of female mice in response to CNO injection (0.3 mg/kg, i.p.) before and after SUS; forced swim test (middle) and locomotor activity (right) of female mice in response to CNO injection after SUS. AAV-DIO-mCherry, *n* = 6; AAV-DIO-hM4Di-mCherry, *n* = 11. *P < 0.05, ***P < 0.001.

We next explored the causal relationship between a reduction in AgRP neuron activity and unpredictable stress-induced depressive behaviors. Mice exposed to 3 days of unpredictable stress did not show significant behavioral changes in sucrose preference or forced swim tests compared with control mice (Fig. 4a; male: Mann Whitney test, *P* = 0.720; female: unpaired *t*-test with the Welch’s correction, *P* = 0.247). Thus, this paradigm was employed as a subthreshold unpredictable stress model in this study. In line with the unaltered behavioral effects, the firing rate and membrane potential of AgRP neurons remained unchanged after exposure to subthreshold unpredictable stress (Fig. 4b; firing rate: Mann Whitney test, *P* = 0.811; membrane potential: *t*_(79)_ = 0.035, *P* = 0.972).

To test whether inhibition of AgRP neurons increases susceptibility to unpredictable stress, AAV vectors expressing Cre-dependent hM4Di and mCherry (AAV-DIO-hM4Di-mCherry and AAV-DIO-mCherry) were injected bilaterally into the ARC of *AgRP-ires-Cre* mice (Fig. 4c). Whole-cell patch-clamp electrophysiological recordings verified the inhibitory effect of CNO on AgRP neurons expressing AAV-DIO-hM4Di (Fig. 4d; paired *t*-test, *t*_(2)_ = 14.62, *P* = 0.005). Sucrose preference was tested in stress-naïve, mCherry- and hM4Di-expressing mice 30 min after a single injection of CNO (0.3 mg/kg, i.p.) and showed no differences between two treatment groups in both sexes (Fig. 4f, male: Mann Whitney test: *P* = 0.320; Fig. 4g, female: *t*_(15)_ = 0.468, *P* = 0.647). After exposure to subthreshold unpredictable stress, male and female mice were tested again 30 min following a CNO injection (Fig. 4e). In male hM4Di-expressing mice, inhibition of AgRP neurons by CNO decreased sucrose preference (Fig. 4f, *t*_(15)_ = 2.828, *P* = 0.013), but caused no significant changes in immobility time in the forced swim test or locomotor activity in the open field (Fig. 4f; FST: *t*_(15)_ = 1.535, *P* = 0.146; locomotor activity: *t*_(15)_ = 1.151, *P* = 0.268). A similar reduction in sucrose preference was observed in female hM4Di-expressing mice after CNO treatment (Fig. 4g, *t*_(15)_ = 4.299, *P* < 0.001). Female mice also exhibited increased immobility time in the forced swim test (Fig. 4g, *t*_(14)_ = 2.877, *P* = 0.012) without significant changes in locomotor activity (Fig. 4g, *t*_(15)_ = 1.572, *P* = 0.137), suggesting a specific effect on despair behavior rather than a general decrease in locomotion. These results indicate that inhibition of AgRP neurons increases susceptibility to stress-induced depression-related behaviors.

### Chemogenetic stimulation of AgRP neurons is sufficient to reverse anhedonia and despair behavior induced by chronic unpredictable stress

**Fig. 5:**
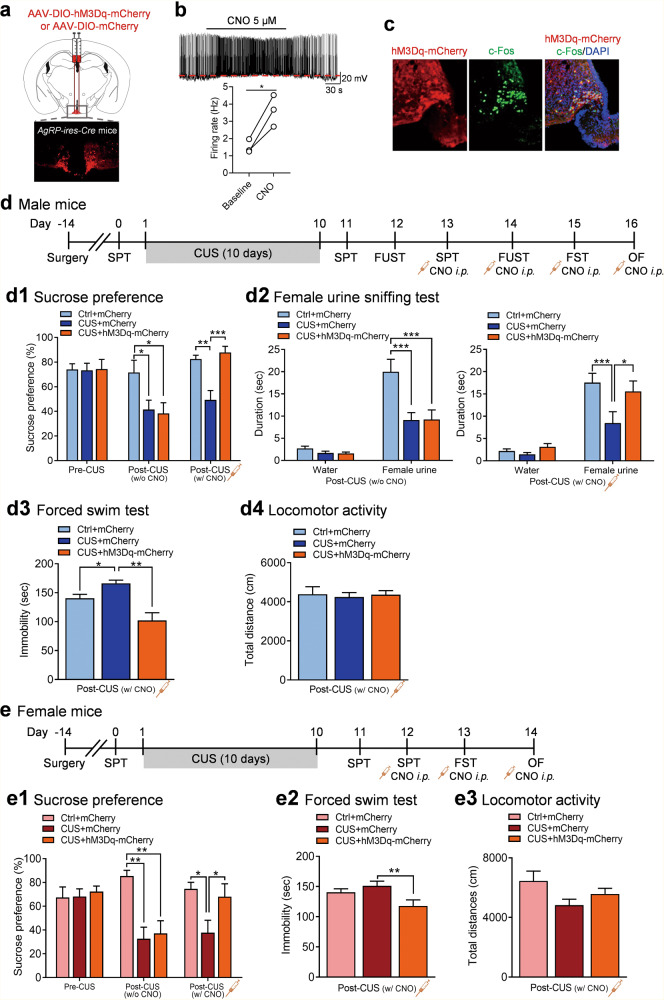
Effects of chemogenetic activation of AgRP neurons on chronic unpredictable stress-induced depressive behaviors. **a** Schematic illustration showing stereotaxic injection of AAV-DIO-hM3Dq-mCherry or AAV-DIO-mCherry in the arcuate nucleus (ARC) and the representative image showing mCherry labeled AgRP neurons in the ARC of *AgRP-ires-Cre* mice. **b** Top, representative traces of action potentials recorded in AgRP neurons infected with AAV-DIO-hM3Dq-mCherry in response to bath application of CNO (5 µM); bottom, firing rates before and after CNO application. **c** Representative images showing CNO induced c-Fos expression (green) in AAV-DIO-hM3Dq-mCherry-infected AgRP neurons (red). **d** Experimental timeline for male *AgRP-ires-Cre* mice. **d1** Sucrose preference test before and after chronic unpredictable stress (CUS). **d2** Female urine sniffing test after CUS without (left) or with CNO injection (right). **d3** Forced swim test. **d4** Locomotor activity. Ctrl + mCherry, *n* = 11; CUS + mCherry, *n* = 14; CUS + hM3Dq-mCherry, *n* = 10. **e** Experimental timeline for female *AgRP-ires-Cre* mice. **e1** Sucrose preference test before and after CUS. **e2** Forced swim test. **e3** Locomotor activity. Ctrl + mCherry, *n* = 12; CUS + mCherry, *n* = 12; CUS + hM3Dq-mCherry, *n* = 14. *P < 0.05, **P < 0.01, ***P < 0.001.

Since suppressing AgRP neuron activity increases susceptibility to subthreshold unpredictable stress, we next asked whether stimulating AgRP neurons can reverse depression-related behaviors induced by chronic unpredictable stress. AAV vectors expressing Cre-dependent hM3Dq or mCherry (AAV-DIO-hM3Dq-mCherry and AAV-DIO-mCherry) were injected bilaterally into the ARC of *AgRP-ires-Cre* mice (Fig. 5a). Electrophysiological recordings verified the stimulatory effect of CNO on AgRP neurons expressing AAV-DIO-hM3Dq (Fig. 5b; paired *t*-test, *t*_(2)_ = 5.649, *P* = 0.030). To confirm the effectiveness of CNO-induced activation of AgRP neurons in vivo, *Agrp-ires-Cre* mice were sacrificed 2 h after CNO injection (0.3 mg/kg, i.p.) and brain sections were processed for immunohistochemical staining. Induction of c-Fos immunoreactivity was observed in the ARC of hM3Dq-expressing mice (Fig. 5c), indicating CNO-induced activation of AgRP neurons. Sucrose preference was reduced after exposure to chronic unpredictable stress in both mCherry- and hM3Dq-expressing male mice prior to CNO treatment (Fig. 5d1; pre-CUS: Kruskal–Wallis test, *P* = 0.803; post-CUS: Kruskal–Wallis test, *P* = 0.022). Stimulation of AgRP neurons by CNO (0.3 mg/kg, i.p.) reversed the reduced sucrose preference in male mice expressing hM3Dq (Kruskal–Wallis test, *P* < 0.001). To examine whether activation of AgRP neurons can alleviate sex-related reward-seeking behavior, the female urine sniffing test was performed in male mice before and after CNO treatment. Consistent with the findings in wild-type mice, female urine sniffing time was decreased in both mCherry- and hM3Dq-expressing male mice after chronic unpredictable stress (Fig. 5d2, left; vector: *F*_(2,64)_ = 9.218, *P* < 0.001; sniffing object: *F*_(1,64)_ = 68.95, *P* < 0.001; vector × sniffing object: *F*_(2,64)_ = 6.162, *P* = 0.004). This effect was reversed by stimulation of AgRP neurons with CNO injection in hM3Dq-expressing mice (Fig. 5d2, right; vector: *F*_(2,64)_ = 5.312, *P* = 0.007; sniffing object: *F*_(1,64)_ = 66.38, *P* < 0.001; vector × sniffing object: *F*_(2,64)_ = 3.228, *P* = 0.046). In addition, the increased immobility time in the forced swim test was reversed by CNO injection in hM3Dq-expressing male mice (Fig. 5d3, Brown–Forsythe ANOVA test, *P* < 0.001), whereas locomotor activity was not altered by either chronic stress or CNO treatment (Fig. 5d4, one-way ANOVA, *F*_(2,32)_ = 0.078, *P* = 0.926). In female mice, similar anti-depressive effects were observed after stimulating AgRP neurons. The decreased sucrose preference was reversed in hM3Dq-expressing female mice after CNO injection (Fig. 5e1; pre-CUS: one-way ANOVA, *F*_(2,37)_ = 0.161, *P* = 0.852; post-CUS without CNO: Kruskal–Wallis test, *P* = 0.001; post-CUS with CNO: Kruskal–Wallis test, *P* = 0.040). Immobility time in the forced swim test was decreased by stimulation of AgRP neurons (Fig. 5e2, one-way ANOVA, *F*_(2,36)_ = 4.458, *P* = 0.019), while no differences in locomotor activity were observed between three treatment groups (Fig. 5e3; one-way ANOVA, *F*_(2,37)_ = 2.780, *P* = 0.075). These results suggest that chemogenetic activation of AgRP neurons is sufficient to reverse depression-related behaviors induced by chronic unpredictable stress.

Since the experiments described above involved multiple injections of CNO, we examined whether chronic, multiple CNO injections could cause overstimulation and subsequent neuronal cell death. *Agrp-ires-Cre* mice received an injection of AAV-DIO-hM3Dq-mCherry on one side and a sham injection on the other side. Three weeks after surgery, mice were given six injections of CNO (0.3 mg/kg, i.p.; once daily). Immunohistochemical staining revealed that the density of AgRP-positive neurons on the side injected with AAV-DIO-hM3Dq was comparable to the sham-injected side (Supplementary Fig. 1), indicating that activation of AgRP neurons by multiple CNO treatments at a relatively low dose used in this study did not cause neuronal cell death.

## Discussion

The present study establishes that AgRP neurons in the ARC are a key component of the neural circuitry underlying depression-related behaviors. Chronic exposure to unpredictable stress caused AgRP neuron dysfunction, as indicated by decreased firing rate and increased firing irregularity of AgRP neurons. These changes were associated with enhanced inhibitory synaptic transmission and reduced intrinsic neuronal excitability. Chemogenetic inhibition of AgRP neurons increased susceptibility to subthreshold unpredictable stress. Remarkably, activating these neurons was sufficient to reverse chronic unpredictable stress-induced anhedonia and despair behavior. These data indicate for the first time that chronic stress induces maladaptive synaptic and intrinsic plasticity of AgRP neurons, which may contribute to the development of depression. Our findings suggest that promoting AgRP neuronal activity may lead to a novel and effective treatment for depression.

Research efforts aimed at understanding the neural circuits underlying depression have been mainly devoted to the prefrontal cortex, hippocampus, and mesolimbic structures [12, 55, 56]. Along with depressed mood, anhedonia is one of the two main diagnostic criteria for major depression [2]. A number of studies have shown that AgRP neurons in the ARC of the hypothalamus contribute to reward-driven feeding [28] and food-seeking behavior [26, 27]. We hypothesized that AgRP neurons participate in regulating reward processing in the context of chronic stress. In this study, we used a chronic unpredictable stress paradigm, which effectively induces anhedonia along with other depression-related behaviors [34–36], including reduced sensitivity to sucrose reward, decreased sex-related reward-seeking behavior, and despair behavior. Correlating with depression-related behaviors, AgRP neuron firing was suppressed in this chronic stress model. The dampened AgRP neuron activity was only observed after a full course of chronic exposure (i.e., 10 days) to unpredictable stress, but not after short-term subthreshold exposure (i.e., 3 days). The impact of stress on AgRP neuron firing seems to be dependent not only upon the type, intensity, and duration of stress but also upon the patterns of stress exposure and the nature of predictability and controllability of stress. Indeed, contrary to our findings with the chronic unpredictable stress model, a recent study using repeated restraint stress in a predictable fashion failed to show any changes in AgRP neuron activity [29]. Exposure to a homotypic (same) stressor repeatedly in a predictable manner (e.g., at the same time of day) has been used as a model of stress habituation wherein responses to a given stressor dampen after repeated presentations [57]. In contrast, the chronic unpredictable stress paradigm used in our study comprises repeated exposure to heterotypic stressors in an unpredictable fashion that prevents habituation and evokes more severe and persistent stress effects. This paradigm models unanticipated stressful experiences that occur in humans, and thus has been widely used as a valid animal model of depression [34, 36, 52]. Our observations of the temporal correlation between changes in AgRP neuron activity and behavioral deficits in this model suggest that the reduction of AgRP neuron activity may contribute to the development of depression-related behaviors.

Regulation of spontaneous firing of AgRP neurons can be achieved by synaptic and intrinsic mechanisms [54, 58–61]. AgRP neurons receive both excitatory and inhibitory synaptic inputs [62]. Recordings of spontaneous excitatory and inhibitory synaptic currents in AgRP neurons demonstrated that chronic unpredictable stress increased inhibitory synaptic transmission without changing excitatory synaptic transmission. Our results suggest that chronic stress shifts the balance between excitatory and inhibitory neurotransmission toward greater inhibition of AgRP neurons. The amplitude but not the frequency of spontaneous IPSCs was increased by chronic unpredictable stress, suggesting enhanced postsynaptic responsiveness. Spontaneous IPSCs comprise both AP-dependent and independent inhibitory synaptic events. In the presence of tetrodotoxin, a blocker of voltage-gated Na^+^ channels, to prevent AP generation and propagation, the response of postsynaptic currents to a single vesicle of transmitter can be measured. The amplitude of miniature IPSCs in AgRP neurons from chronically stressed mice remained higher in the absence of APs. These data suggest that chronic unpredictable stress may increase inhibitory synaptic transmission onto AgRP neurons partially through augmented postsynaptic sensitivity to synaptically released GABA, due either to increased function or increased number of postsynaptic GABA receptors clustered at synaptic sites [63, 64].

In the absence of synaptic transmission, AgRP neurons were capable of intrinsically firing [65, 66]. We found that chronic unpredictable stress decreased the intrinsic excitability of AgRP neurons. It was noted that AgRP neurons from control mice fired at a slower rate after blocking glutamatergic and GABAergic synaptic transmission (2.9 Hz vs 5.5 Hz in the presence vs absence of synaptic blockers), suggesting a net predominance of excitatory over inhibitory synaptic inputs to AgRP neurons under normal, non-stressed conditions. This predominant excitation is reduced by chronic stress through both synaptic and intrinsic mechanisms.

AgRP neurons from chronically stressed mice fired more slowly and irregularly, with a correlation between the degree of firing irregularity and the firing rate for individual neurons. This phenomenon was observed both in the absence and presence of synaptic blockers. An increase in the irregularity of neuron firing reflects instability [67]. How chronic stress causes AgRP neurons to generate slow and irregular firing is currently unknown, but multiple mechanisms are hypothesized to explain firing irregularity. First, it can arise from fluctuating patterns of synaptic activity [68, 69]. Second, irregular neuronal firing may be caused by intrinsic cellular mechanisms through the stochastic gating of ion channels in the membrane, such as potassium ion channels [70–73]. One principal feedback mechanism in the control of the frequency and patterning of neuronal firing is afterhyperpolarization, or AHP [74–76]. AHP currents are mainly mediated by calcium-dependent potassium channels [77, 78], but also partly by calcium-independent potassium channels [79]. Particularly, small-conductance calcium-activated potassium (SK) channels have been reported to control the spontaneous firing regularity both in vivo and in vitro [80, 81]. AgRP neurons express high levels of SK channels [82]. ATP-sensitive potassium channels were also detected in AgRP neurons [61]. We found that chronic unpredictable stress caused an increase in the amplitude of AHP in AgRP neurons. This could constitute an inhibitory driving force for spontaneous neuronal activity as AHP amplitude is reversely correlated with the firing frequency [83–85]. Chronic unpredictable stress may thus reduce AgRP neuron electrical activity via activation of calcium-dependent and independent potassium channels.

Importantly, this study demonstrates that chemogenetic inhibition of AgRP neurons increased susceptibility to subthreshold unpredictable stress. Conversely, activation of AgRP neurons was able to reverse chronic unpredictable stress-induced depression-related behaviors. These results suggest that AgRP neuronal activity may be necessary and sufficient for the manifestation of depression-related behaviors. As AgRP neurons project to multiple brain regions involved in stress reactivity and reward processing [20, 62, 86], it is not clear which downstream targets may be contributing to the effects of AgRP neurons on depression-related behaviors. However, available anatomical evidence suggests that AgRP neurons may functionally interact with the mesolimbic pathway [20, 62, 86], which has been implicated in depression [56]. The mesolimbic dopamine system, composed of dopamine neurons of the ventral tegmental area (VTA) and their projections to the nucleus accumbens (NAc), is most often associated with the rewarding effects of food and sex and plays an essential role in stress-induced anhedonia and other depression-related behavioral abnormalities [87, 88]. Selective inhibition of VTA dopamine neurons induces anhedonia and behavioral despair; conversely, phasic activation of VTA dopamine neurons reverses chronic mild/unpredictable stress-induced depression-like phenotypes, a phenomenon dependent on dopamine transmission in the NAc [41]. AgRP neurons in the ARC have been shown to innervate the VTA and play a role in setting VTA dopamine neuronal activity and determining reward-associated behaviors [89]. Another candidate downstream target of AgRP neurons is the bed nucleus of the stria terminalis (BNST) [20, 62], which has emerged as a key player in stress-related disorders [90, 91]. Lesions of this nucleus alter despair behavior [92] and block the behavioral consequences of stress [93]. Future studies will examine whether stimulation or inhibition of AgRP neurons can influence the activity of VTA and BNST neurons in the chronic unpredictable stress model and identify which neuronal populations mediate the effects of AgRP neurons on depression-related behaviors.

## Supplementary information

SUPPLEMENTAL MATERIAL
