# Supplementary material for: Chronic unpredictable stress induces depression-related behaviors by suppressing AgRP neuron activity
Source: Mol Psychiatry. 2021 Jan 11;26(6):2299–315. doi: 10.1038/s41380-020-01004-x (PMC8272726; doi:10.1038/s41380-020-01004-x)
Supplement: Supplementary file 1 — SUPPLEMENTAL MATERIAL [file 41380_2020_1004_MOESM1_ESM.docx]

**Supplementary Information**


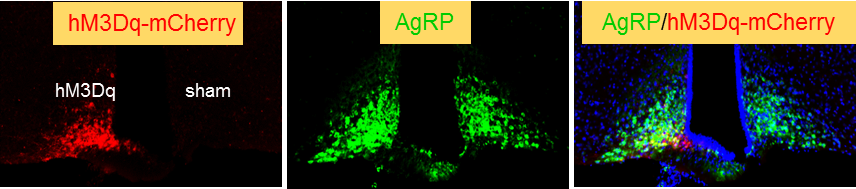


**Supplementary Fig. 1.** Representative images showing AAV-DIO-hM3Dq-mCherry injection (left, red), AgRP immunofluorescence staining (middle, green), and merged image (right; DAPI, blue) in the ARC of *Agrp-ires-Cre* mice that received 6 CNO injections (0.3 mg/Kg, i.p., daily).
